# Nano Carbon Black-Based High Performance Wearable Pressure Sensors

**DOI:** 10.3390/nano10040664

**Published:** 2020-04-02

**Authors:** Junsong Hu, Junsheng Yu, Ying Li, Xiaoqing Liao, Xingwu Yan, Lu Li

**Affiliations:** 1 State Key Laboratory of Electronic Thin Films and Integrated Devices, School of Optoelectronic Science and Engineering, University of Electronic Science and Technology of China (UESTC), Jianshe North Road, Chengdu 610054, China; uestchujunsong@163.com (J.H.); jsyu@uestc.edu.cn (J.Y.); 2 Research Institute for New Materials Technology, Chongqing University of Arts and Sciences, Honghe Avenue, Chongqing 402160, China; xiaoqin5122@163.com (X.L.); yan_xing_wu@126.com (X.Y.)

**Keywords:** nano carbon black, polydimethylsiloxane, pressure sensors, wearable electronics

## Abstract

The reasonable design pattern of flexible pressure sensors with excellent performance and prominent features including high sensitivity and a relatively wide workable linear range has attracted significant attention owing to their potential application in the advanced wearable electronics and artificial intelligence fields. Herein, nano carbon black from kerosene soot, an atmospheric pollutant generated during the insufficient burning of hydrocarbon fuels, was utilized as the conductive material with a bottom interdigitated textile electrode screen printed using silver paste to construct a piezoresistive pressure sensor with prominent performance. Owing to the distinct loose porous structure, the lumpy surface roughness of the fabric electrodes, and the softness of polydimethylsiloxane, the piezoresistive pressure sensor exhibited superior detection performance, including high sensitivity (31.63 kPa^−1^ within the range of 0–2 kPa), a relatively large feasible range (0–15 kPa), a low detection limit (2.26 pa), and a rapid response time (15 ms). Thus, these sensors act as outstanding candidates for detecting the human physiological signal and large-scale limb movement, showing their broad range of application prospects in the advanced wearable electronics field.

## 1. Introduction

Electronics with flexible, extensible, and wearable traits have recently attracted significant research interests for a broad range of applications, including electronic skins [1,2], health-monitoring devices [3,4,5,6], flexible displays, and energy-harvesting devices [7]. Wearable pressure sensors [8,9], as a significant sub-area of wearable electronics, should have stable mechanical compliance. They should be able to comply with natural motions to monitor personal activities and human health effectively. For practical applications, pressure sensors should have ultra-high sensitivity, be quite flexible, and be relatively stable. So far, four types of pressure sensors including piezoresistive [10,11,12,13,14,15,16], capacitive [17,18,19], piezoelectric [20,21,22], and triboelectric [23] have been reported. In particular, piezoresistive pressure sensors are extensively used, ascribed to their simple and facile fabrication process, superior sensitivity, and excellent response mechanism. By converting subtle mechanical deformation into the variation in resistance of the briskly conducting materials, piezoresistive sensors can easily detect various mechanical deformation loadings. Nonetheless, a majority of the reported sensors may not simultaneously achieve pressure sensing ability with excellent sensitivity and a wide workable range, which restrict their practical applications. Therefore, it is extraordinarily desired to investigate multifunctional sensing platforms with both ultra-high sensitivity and a wide workable pressure range through a cost-effective and simple fabrication process.

In general, flexible piezoresistive-type sensors consist of the following two dominating components: flexible substrates and compatible conductive and active layers. In practical applications, polydimethylsiloxane (PDMS) [24,25] films are extensively used as the flexible substrates to assemble with compatible material. The choice of the conductive layer plays a dominant role. So far, a large variety of materials including carbon nanotubes [26], graphene [27,28,29], metal material nanoparticles/nanowires (for example, AgNPs and AgNWs) [30,31], and environmentally-friendly organic conductive polymer [32,33] have been utilized in piezoresistive-type sensors. Carbon-based materials from unprocessed materials have received far-ranging interest due to their prominent electrical conductivity, cost effectiveness, and large-scale production capability. Furthermore, carbonized silk, cotton [34], corncobs, and mushrooms have been constructed as sensing components for wearable strain sensors. The carbonation process usually takes place at a high temperature in an atmosphere of mixed argon and hydrogen in a tubular furnace.

As a traditional sensing material, carbon black possesses the advantages of low cost, easy production, natural abundance, and favorable conductivity and has been widely used as a building phase for the construction of conductive polymer composites [35,36,37,38]. With an appropriate proportion of carbon black, these composites possess flexibility and piezoresistivity, making them suitable sensing materials for flexible strain sensors. The mechanism of electrical conduction in these composites is the formation of a continuous network of conductive carbon black throughout the insulating polymer matrix. The level of electrical conductivity in these heterogeneous materials depends primarily on the concentration and geometry of the carbon black filler. However, for these conventional carbon black fillers, a rather high loading is required to achieve satisfactory electrical properties, resulting in material redundancy and detrimental mechanical and sensing properties [39,40,41,42].

In this study, a novel nano carbon black (NCB) ultra-thin coating on PDMS was employed as the sensing materials for wearable pressure sensors. The NCB coating was deposited by collecting kerosene soot on the surface of a glass base and then transferred to a flexible PDMS substrate. Kerosene soot is an atmospheric pollutant generated during the insufficient burning of hydrocarbon fuels. The NCB particles in the ultra-thin coating were connected with each other to form a continuous network, effectively avoiding deterioration of the mechanical properties of the PDMS to which higher filler concentrations may lead [36,39]. The conductivity of this obtained ultra-thin NCB coating was higher than those of polymer composites filled with carbon black [39,40,41,42]. Flexible pressure sensors were constructed with an upper bridge of NCB-coated PDMS and a bottom interdigitated textile electrode screen printed with silver (Ag) paste. Owing to the high conductivity of the NCB coating, the large surface roughness of the fabric electrodes, and the softness of PDMS substrate, the piezoresistive pressure sensor prepared herein exhibited excellent performance, including ultra-high sensitivity (31.63 kPa^−1^ within the range of 0–2 kPa), a large feasible pressure range (0–15 kPa), a low detection limit (2.26 pa), and a rapid response (15 ms), which are among the best outcomes for wearable electronics. On account of these outstanding detection properties, these electronic sensors were able to detect wrist pulse and carotid pulse signals. The concept paves a novel way for the cost-effective, lightweight, and simple fabrication of wearable electronics.

## 2. Results and Discussion

### 2.1. Fabrication of the Nano Carbon Black-Based Sensor

**Figure 1:**
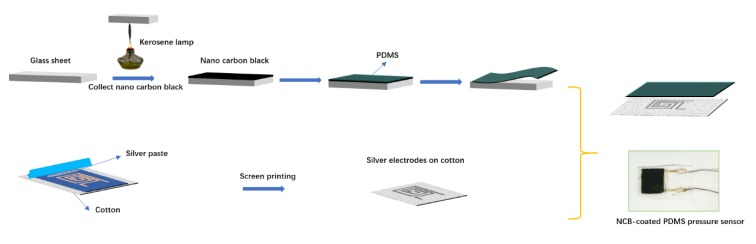
Schematic illustration of the fabrication process and device structure of the nano carbon black (NCB)-coated piezoresistive-based sensor and its digital photograph.

Figure 1 presents a schematic illustration of the fabrication process of the NCB-based sensor. A piece of glass was rinsed with acetone and deionized water. NCB was coated on the surface of the glass by collecting the soot from a burning kerosene lamp. PDMS was deposited on the surface of the carbon black as a uniform film by the drop casting method followed by solidification in an oven at 70 °C for 4 h. After removal from the glass sheet, a conductive black carbon film based on PDMS was obtained with a typical resistance of 2.03 kΩ sq^−1^.

The flexible bottom electrodes were fabricated on fabric by screen printing technology (Figure 1). After scraping, the Ag paste traversed the designed screen mesh and then was printed on the fabric substrate. After exsiccation at 80 °C for 20 min, Ag electrodes with an interdigitated configuration and an ultra-high conductivity (the sheet resistivity was approximately 0.37 Ω sq^−1^) were coated on the fabric substrate. Further, a slice of NCB-coated PDMS was used to cover the upper surface of the Ag-coated electrodes, and then, a thin layer of VHB tape was used to encapsulate it, while maintaining mechanical rebound resilience properties. The devices obtained by the above-mentioned fabrication process were flexible owing to the elastic nature of PDMS and fabric. Moreover, screen printing (roll-to-roll) is compatible with industrial processes, which is also suitable for a cost-effective, simple, and large-scale synthesis approach.

### 2.2. Morphological Characteristics of Nano Carbon Black and Silver Electrodes 

**Figure 2:**
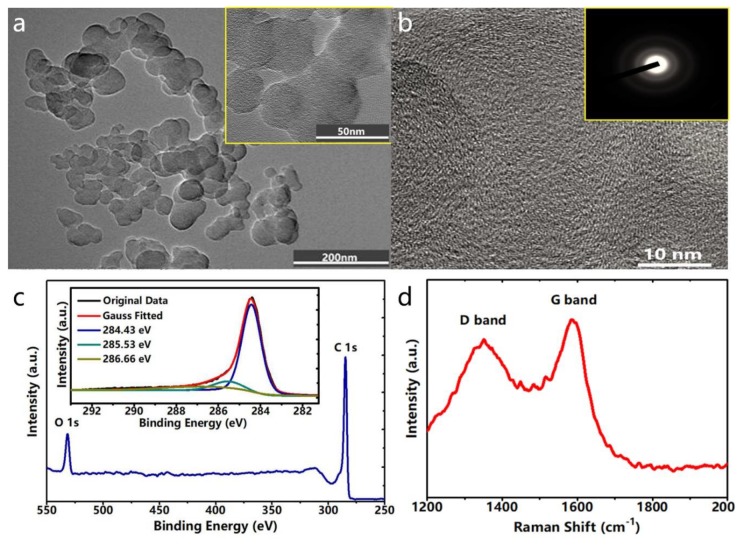
(**a**) TEM image of NCB and its higher magnification (inset). (**b**) High-resolution TEM image of the NCB and its SAED pattern (inset). (**c**) XPS spectrum showing the O 1s and C 1s peaks of the NCB. The inset in the panel shows the deconvolution of the C 1s XPS peak. (**d**) Raman spectrum of NCB.

Furthermore, the composition of the NCB coating was analyzed. Figure 2a exhibits transmission electron microscopy (TEM) images of NCB, elucidating that NCB was composed of spherical nanoparticles with a particle size ranging from 20 to 50 nm. The higher magnification in the illustration shows that carbon nanospheres were joined by weak intermolecular interactions [43,44]. Moreover, Figure 2b depicts that several crystalline carbon nanospheres together with amorphous carbon were clearly recognized in the NCB as further confirmed by selected area electron diffraction (SAED) imaging. The SAED pattern showed that a distorted lattice fringe belonged to the (002) plane diffraction ring of hexagonal graphite [45,46].

Elemental analysis of the soot from the kerosene lamp observed by X-ray photoelectron spectroscopy (XPS) more deeply exposed the contents of C and O to be 90.54% and 9.46%, respectively. The outcomes elucidated that the acquired NCB did not include other elements rooted in organic contaminates. Figure 2c shows that the C 1s spectrum of the NCB was fitted with three correlated peaks at 284.4 eV (attributed to sp^2^ hybridized C=C), 285.5 eV (attributed to sp^3^ hybridized C–C), and 286.7 eV (attributed to sp^3^ hybridized C–O), elucidating a comparatively high degree of graphitization. This result was further confirmed by Raman spectroscopy. Figure 2c depicts the existence of a D band associated with the amorphous carbon at 1350 cm^−1^ and a G band at 1587 cm^−1^ closely connected with the E_2g_ mode of crystalline carbon due to the vibration of sp^2^-bonded carbon atoms in a two-dimensional (2D) hexagonal lattice. The existence of the G band manifested that the NCB was composed of highly ordered pyrolytic graphite [47].

**Figure 3:**
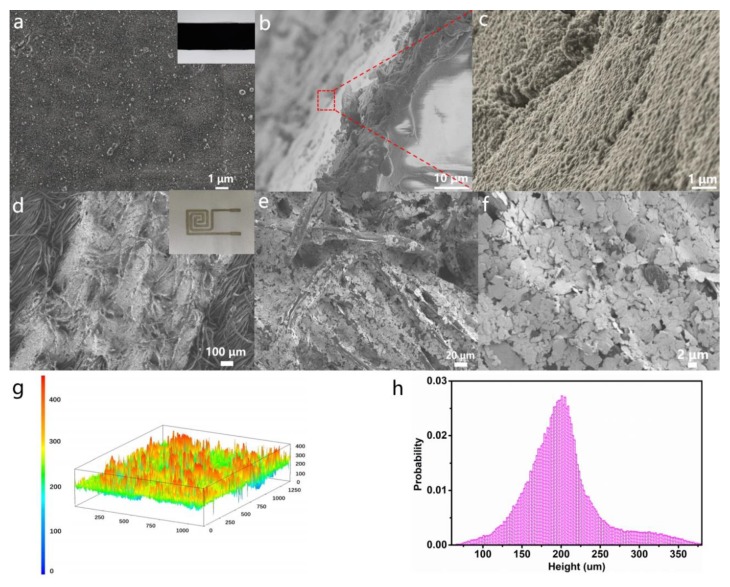
Morphological features of the NCB-coated PDMS surface. (**a**) A plane view of the SEM image of NCB-coated PDMS and its digital inset photograph. (**b**,**c**) Cross-sectional SEM images of the edge portion of NCB-coated PDMS under different magnifications. (**d**) SEM image of screen printed electrodes and the inset showing its digital photograph. (**e**,**f**) SEM image of the Ag electrode screen printed on textile under different magnifications. (**g**) 3D morphology of the Ag electrode-coated fabric. (**h**) The probability distribution of the surface heights.

Figure 3a shows the SEM image of the NCB-coated PDMS, exhibiting a very smooth surface with only a few scattered particles. A digital photograph of the NCB-coated PDMS is also shown in the inset of Figure 3a. A cross-sectional SEM image (Figure 3b) of PDMS covered with NCB showed that the thickness of the NCB layer was about 10 μm. At a higher magnification, the fluffy uniform structure of NCB wrapped around the surface of the PDMS was observed, as shown in Figure 3c.

Figure 3d demonstrates that by employing the screen printing technology, a clear insulation gap with a minimum gap size of only 430 μm between two adjoining Ag electrodes could be easily obtained, while maintaining the inherent weave characteristics of the original fabric. The inset in Figure 3d shows a digital photograph of the Ag electrodes printed on cotton. The SEM image (Figure 3e) shows that each individual fiber was wrapped with a tight Ag layer. Owing to the porous structure of the fabric, the Ag paste was able to penetrate the surface of the inner fiber. The detection performance of the pressure sensor depended on its microstructure. The decorated Ag fabric exhibited a multi-line interwoven microstructure that created more conductive loops when pressure was applied. The high-magnification SEM image indicated that the Ag paste was tightly wound around the surface of the fiber (Figure 3f), and the accumulated morphology further led to the increase in the roughness [48]. 

Moreover, for Ag paste-coated conductors with a non-uniform height distribution, the nonwoven fabric substrate exhibited 3D confocal imaging results in the range of 150–250 μm (Figure 3g). The probability distribution of the electrode surface height (Figure 3h) indicated that the surface height was stochastic and comparatively close to a Gaussian distribution centered at 200 μm. Such a randomly distributed surface of the electrode was beneficial for the linear increase of the device.

In the absence of pressure, there were only a few contact points between the conductive NCB and the Ag electrode. When appropriate external pressure was applied, the surface of the NCB-coated PDMS became deformed, and the common contact area and immediate current transmission path between these NCB-coated PDMS and the Ag fabric electrode underwent a sudden increase [49].

### 2.3. Electromechanical Performance of the Nano Carbon Black Pressure Sensor

**Figure 4:**
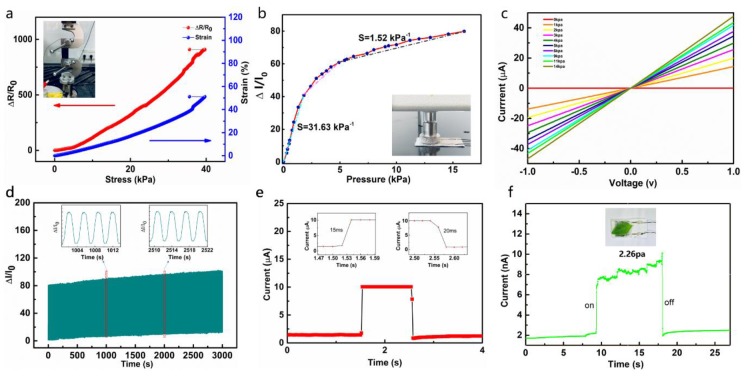
Basic electromechanical sensing performance: (**a**) Relative change in resistance and strain when the NCB-coated PDMS is pulled up and inset image of its measuring equipment. (**b**) Relative change in the current of the pressure sensing when the pressure increases from 0 to 15 kPa and inset image of its measuring equipment. (**c**) I–V curves under different pressures. (**d**) The circulation testing of the NCB sensor with applied pressure of 15 kPa. (**e**) Response/release time of the NCB sensor under the pressure of 20 Pa. (**f**) The loading and shift of a leaf on the NCB sensor response to current; the corresponding applied pressure is merely 2.26 Pa.

Owing to the recoverable deformation of the NCB-coated PDMS within a certain range, the tensile strength of the NCB-coated PDMS was measured to explore its tensile properties. The red curve in Figure 4a depicts the stress and relative resistance change function. The blue curve is a function of the stress-induced range of variation from 0 to 50%. When the stress reached 40 kPa, both curves showed the existence of obvious inflexion points, which proved that the NCB-coated PDMS was destroyed when the deformation variable reached about 53%. The change was relatively proportional to the applied strain force in the range of 0–50%; the linearity was very high; and the GF was as high as 20.97 in the range of 5–35% deformation. GF is defined as GF = R − R_0_/Rλ, where λ is the strain and R_0_ denotes the initial electrical resistance without applied strain. Within such a large deformation range, the obtained value of GF = 20.97 (Figure S1) based on all carbon black was better than the traditional metal strain gauge (GF = 2, λ < 5%). Although previously reported polyimide strain sensors also reached fairly high values of GF, they were usually limited to very low stretch (λ < 5%), thus restricting their application to human motion monitoring.

The SourceMeter (Keithley 2400, Beaverton, OR, USA) was used to investigate the electrical properties of pressure sensing capacity with NCB as the active layer and a PDMS substrate. Figure 4b and Figure S2 exhibit a monotonic increase in relative resistance change observed under a pressure range of 0 to 15 kPa. Noteworthy is that the sensitivity (δ(ΔI/I_0_)/δP, where ΔI denotes the relative variation of current, I_0_ represents the initial current without applied pressure, and δP is the change in applied pressure) based on NCB showed superior performance compared to the recently reported pressure sensors. Herein, the performance of the NCB sensor was explored under different pressures. Figure 4b shows three relatively linear parts: 0–2, 2–5, and 5–15 kPa, with S values of 31.63, 5.04, and 1.52 kPa^−1^, respectively, indicating both ultra-high sensitivity and a wide workable range. The high sensitivity and wide workable range of our devices may be due to the excellent mechanical and structural properties of the NCB-based pressure sensors. The substrates we used, PDMS and cotton fabric, had a low compressive effective elastic modulus. The current switching behavior of our sensors could be attributed to the fact that the OFF-state was initially at a break insulating condition; when applying pressures, a high ON-state current flow could be attained by the conductive NCB-decorated PDMS that bridged the two interdigitated silver electrodes. The degree of increase in contact area with applied pressure depended on the elastic modulus of the sensing elements. The large deformation of NCB-decorated PDMS with low elastic moduli produced a large increase in contact area. A surface structure of the bottom fabric electrode with a wide size distribution was also proposed to improve the sensitivity and working range.

Figure 4c shows that the current–voltage (I–V) curve under various pressures increased linearly within the appropriate voltage range of −1 to 1 V, indicating that the NCB sensor complied with Ohm’s law. Furthermore, the stability of flexible sensors under the pressure of 2.5 kPa and frequency of 0.5 Hz was also investigated. The outcomes indicated that the current amplitude almost remained unchanged after approximately 1500 cycles of repeated loading/unloading (Figure 4d).

We also measured the relative electrical current variations of the NCB sensor under the pressures of 0 kPa–15 kPa–0 kPa. The local inelastic deformation process that always exists in textile materials explained the electrical hysteresis of the device (Figure S1b), similar to many other piezoresistive-type pressure sensors. Moreover, the sensor prepared herein showed a rapid response (15 and 20 ms, respectively) with an instantaneous pressure of 400 Pa (Figure 4e). Figure 4f shows that the pressure sensor device was extraordinarily sensitive when placing and removing very light objects such as leaves, corresponding to a pressure of 2.26 Pa.

### 2.4. Monitoring of Human Physiology

**Figure 5:**
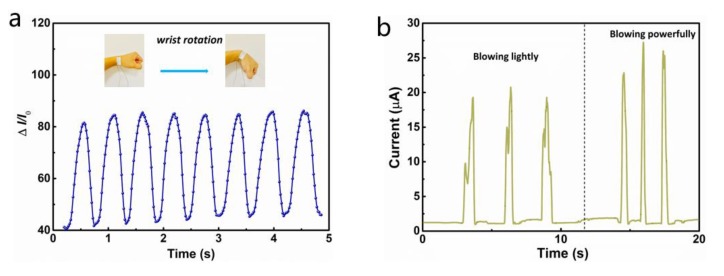

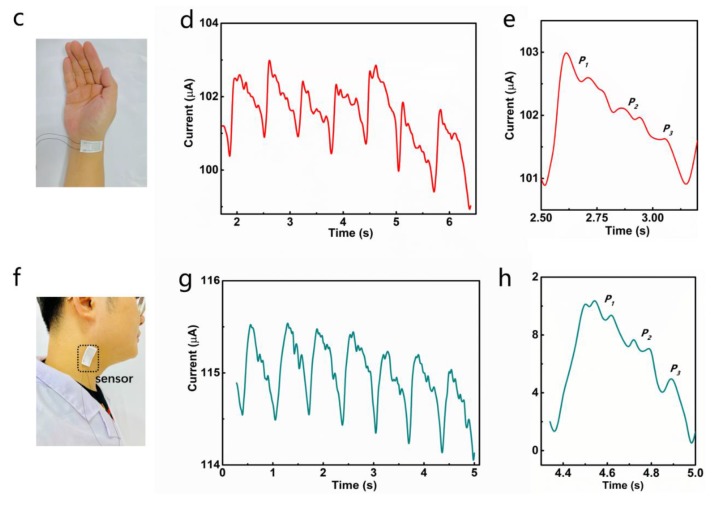
Real-time detection of different electrical signals using the NCB-coated PDMS pressure sensor: (**a**) The response of relative current changes caused by rapid whirling of the wrist. (**b**) The current signal for detecting different strengths of gas generated from the mouth. (**c**) Photograph of a sensor mounted on the wrist for pulse detection. (**d**,**e**) A wrist pulse waveform and a single pulse waveform recorded by the NCB sensor. (**f**) An optical image of the NCB-coated PDMS sensor attached to the neck for arterial pulse waves’ detection. (**g**) Neck pulse waveform of the test sensor and (**h**) a single pulse waveform.

Based on its outstanding performance, we further demonstrated the real-time application of NCB sensors and in situ biomedical testing. Figure 5a shows the real-time response of the wrist when the NCB sensor was attached to the wrist as it rotated rapidly and also the stability of the NCB sensor exhibited by the wrist at high speed. Moreover, the data from Figure 5b also show that these wearable sensors could be used to detect breathing, which is a critical part of real-time monitoring of physical health. Owing to the ultra-high sensitivity, this equipment could differentiate the gas strength from the mouth. When the gas generated from the mouth reached the surface of the equipment, the sensor was able to record the slight mechanical deformation caused by the gas pressure easily and convert it into the desired signal due to the rapid electronical response and ultra-high sensitivity. Different blow intensities such as “blowing lightly” and “blowing powerfully” could be identified by the NCB sensor, which revealed the existence of prominent sensitivity and unique memory patterns. Each different breath was recorded three times, and similar correlative characteristic peaks and troughs on each curve could be clearly seen, elucidating that the breath detection had fantastic repeatability. Thus, this system also provided a robust and effective method for human health monitoring.

Figure 5d demonstrates that with the aid of scotch tape, a lightweight and a highly sensitive sensor could be conformally attached to the subject’s brachial artery. According to the data obtained (Figure S3), the heart rates of the tested subject under normal conditions and after running for 30 min were 84 and 128 beats min^−1^, respectively. Representative human pulse waveform peaks correlated with “P1” (percussion), “P2” (tidal), and “P3” (diastolic) were obviously distinguished, which was likely to attributed to the conformal contact of the flexible sensor with the skin surface [50].

Furthermore, to further illustrate the reliability of the data, a record of the respiratory frequency that continuously tracked sleep is shown in Figure 5f. Continuous tracking of the respiratory rate of sleep is an effective way to avoid sleep apnea; however, sleep apnea is very likely to be misdiagnosed among numerous emergency diseases. At present, most of the specialized biomedical technologies use expensive, cumbersome, and uncomfortable instruments, which limit an extensive range of practical applications. Our flexible sensors could be mounted on the tester’s neck using transparent tape to capture the rise and fall of the carotid artery, providing a cost-effective and simple approach for monitoring real-time breathing. Figure 5g shows that the sensor installed on the subject’s artery recorded the pulse rate in the normal condition. There were still three peaks, P1, P2, and P3, which were consistent with the results of the previous test.

## 3. Conclusions

The design and fabrication of a high-performance wearable pressure sensor based on nano carbon black (NCB) was demonstrated in this study. The conversion of NCB generated from a kerosene lamp to functional electronics was an easily-fabricated, eco-friendly, and cost-efficient method to turn domestic waste into an extraordinarily useful device. Owing to the distinct loose porous structure and large-scale surface roughness of the textile electrodes and the softness of PDMS, the as-constructed pressure sensor revealed fantastic sensitivity (31.63 kPa^−1^ within the range of 0–2 kPa), rapid response (15 ms), and a large workable pressure sensor range (0–15 kPa). On account of these prominent sensing properties, we illustrated its actual application in detecting numerous desired mechanical signals such as wrist movement, acoustic vibration, and even faint pulses with fantastic repeatability. The study showed that this sensor possessed broad prospects for health monitoring as a flexible wearable electronic device.

## 4. Experimental Section

### 4.1. Preparation of Carbon Black

The kerosene lamp used in this experiment was purchased from the local market. Kerosene was blended with refined straight-run kerosene or hydrocracked kerosene fractions. Its main component included C10–C16 alkane, and it also contained a small number of aromatic hydrocarbons, unsaturated hydrocarbons, cyclic hydrocarbons, and other impurities. A piece of glass (75 × 25 mm) was cleaned with acetone and deionized water prior to the experiment. The black soot formed by burning the kerosene lamp in the air was then used to cover the surface of the glass, i.e., carbon black. However, it was difficult to induce a uniformly dense carbon black layer at the tail of the flame; thus, the center of the glass piece was placed directly above the flame tail of 10 mm. The NCB deposition could be controlled by changing the deposition time.

### 4.2. All Carbon Black Pressure Sensor Fabrication

The PDMS prepolymer (the base monomer and the curing agent were stirred for 5 min in a weight ratio of 10:1) was deposited on the surface of previously coated NCB as a uniform film by drop casting, followed by placing it in an oven at 80 °C for 4 h. After peeling from the glass sheet, the conductive NCB film was covered on the surface of PDMS. For the bottom interdigitated textile electrode, commercially conductive silver paste (ENSON CD-03, Guangzhou, China) was imprinted on pre-washed fabric by the screen printing method. After drying at 80 °C for 25 min, the patterned silver electrodes on the fabric substrate with ultra-high conductivity were obtained. Finally, the bottom of the silver electrode and the top NCB-coated PDMS were encapsulated with VHB film (3M™ VHB™ Tape 4910). The devices were compressed with a stress of 50 kPa before testing.

### 4.3. Device Characterization

Scanning electron microscopy (SEM) was performed using a QUANTA 250 micrometer (GeminiSEM 300, Hallbergmoos, Germany). The TEM images were obtained using a field-emission TEM (FE-TEM, JEOL JEM 2100F, Beijing, China). Raman spectra were performed with a Raman spectroscope (RENISHAW RM2000, Gloucestershire, UK) with a later excitation wavelength of 532 nm. X-ray photoelectron spectroscopy (ThermoFisher K-Alpha, Waltham, MA, USA) was used for elemental analysis of nano carbon black. The 3D morphology of the Ag-coated fabric was characterized by a laser scanning confocal microscope (OPTELICS C130, Kanagawa, Japan). The sensitivity of the NCB pressure sensor was measured using a computer-controlled force gauge (HP-10, China Handpi Instruments, Zhejiang, China) as the pressure source. In order to obtain the resistance of the piezoresistive sensor to multiple stimuli, the resistance and current were obtained using an electrochemical workstation (CHI 760E, Shanghai, China) and a digital source meter (Keithley 2400, Beaverton, OR, USA). The stability of flexible sensors was tested by a fatigue testing machine (Wance EDT 104B, Shenzhen, China) under a pressure of 2.5 kPa at a frequency of 0.4 Hz, and an external electrochemical workstation was connected to test the change of resistance of the sensors.

## Supplementary Materials

The following are available online at https://www.mdpi.com/2079-4991/10/4/664/s1: Figure S1. (a) Relative resistance change as a function of tensile strain. (b) Relative current change as a function of pressure change of 0 kPa-15 kPa-0 kPa, Figure S2. Relative change of the current under pressures of different sensors with or without NCB in the same batch, Figure S3. Arterial pulse waves under normal and after strenuous exercise conditions.
