# Supplementary material for: Nano Carbon Black-Based High Performance Wearable Pressure Sensors
Source: Nanomaterials (Basel). 2020 Apr 2;10(4):664. doi: 10.3390/nano10040664 (PMC7221763; doi:10.3390/nano10040664)
Supplement: Supplementary file 1 [file nanomaterials-10-00664-s001.pdf]

Supporting Information: Nano Carbon Black-Based High Performance Wearable Pressure Sensors

Junsong Hu [1,2], Junsheng Yu [1], Ying Li [2,*], Xiaoqing Liao [2], Xingwu Yan [2] and Lu Li [2,*]

1 State Key Laboratory of Electronic Thin Films and Integrated Devices, School of Optoelectronic Science and Engineering, University of Electronic Science and Technology of China (UESTC), Jianshe North Road, Chengdu 610054, China; uestchujunsong@163.com (J.H.); jsyu@uestc.edu.cn (J.Y.)

2 Research Institute for New Materials Technology, Chongqing University of Arts and Sciences, Honghe Avenue, Chongqing 402160, China; xiaoqin5122@163.com (X.L.); yan_xing_wu@126.com (X.Y.)

* Correspondence: leoyingchem@163.com (Y.L.); lilu25977220@163.com (L.L.)

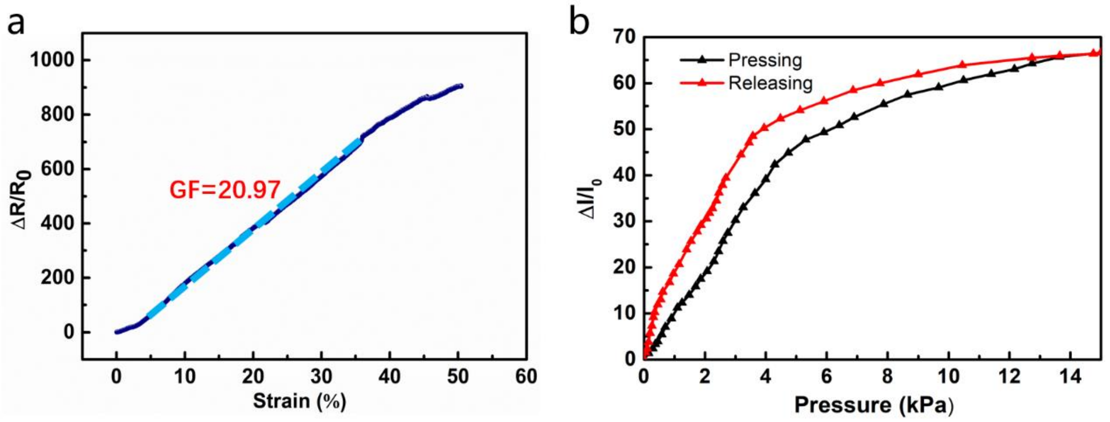

Figure S1. (a) Relative resistance change as a function of tensile strain. (b) Relative current change as a function of pressure change of 0 kPa-15 kPa-0 kPa.

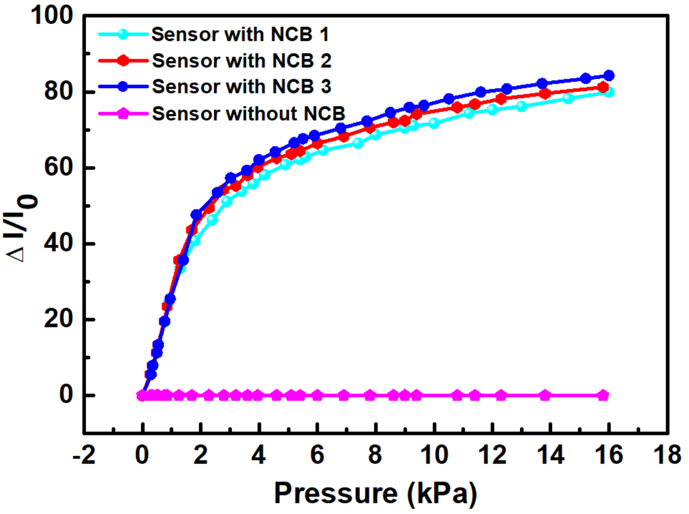

Figure S2. Relative change of the current under pressures of different sensors with or without NCB in the same batch.

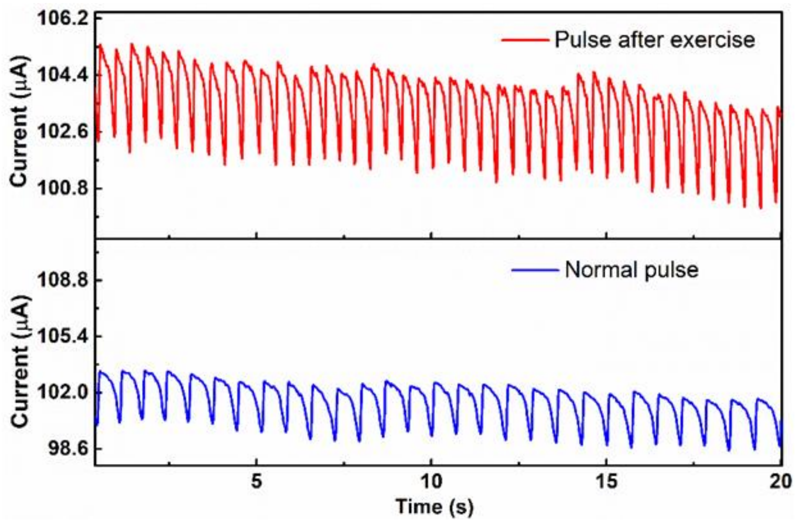

Figure S3. Arterial pulse waves under normal and after strenuous exercise conditions.
